# 5-Chloro-3,6-dimethyl-1-phenyl-1*H*,4*H*-pyrano[2,3-*c*]pyrazol-4-one

**DOI:** 10.1107/S1600536812028528

**Published:** 2012-06-30

**Authors:** Abdullah M. Asiri, Hassan M. Faidallah, Khalid A. Alamry, Seik Weng Ng, Edward R. T. Tiekink

**Affiliations:** a Center of Excellence for Advanced Materials Research (CEAMR), King Abdulaziz University, PO Box 80203, Jeddah 21589, Saudi Arabia; b Chemistry Department, Faculty of Science, King Abdulaziz University, PO Box 80203, Jeddah 21589, Saudi Arabia; c Department of Chemistry, University of Malaya, 50603 Kuala Lumpur, Malaysia

## Abstract

In the title compound, C_14_H_11_ClN_2_O_2_, two independent mol­ecules (*A* and *B*) comprise the asymmetric unit with the main difference between them being the relative orientation of the pendent phenyl ring with respect to the fused-ring system [dihedral angles = 8.32 (8)° (*A*) and 28.32 (8)° (*B*)]. In the crystal, the *A* mol­ecules are connected into a linear supra­molecular chain along the *a* axis *via* C—H⋯O inter­actions and linked to this *via* C—H⋯Cl inter­actions are the *B* mol­ecules. The chains are connected into layers in the *ab* plane by π–π inter­actions between pyrazole (*A*) and pyran (*B*) rings, and between pyrazole (*B*) and pyran (*A*) rings [centroid–centroid distances = 3.5442 (11) and 3.4022 (10) Å, respectively].

## Related literature

For the analgesic and anti-inflammatory activity of pyrano[2,3-*c*]pyrazole derivatives, see: Kuo *et al.* (1984 ▶). For the synthesis, see: Gelin *et al.* (1983 ▶). For the structure of the derivative without a chloro substituent, see: Asiri *et al.* (2012 ▶).
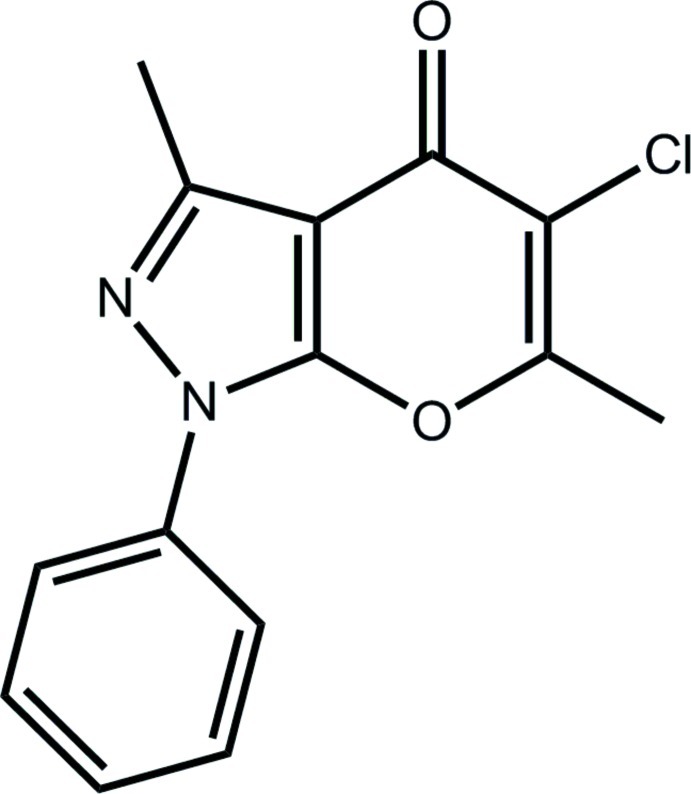


## Experimental

### 

#### Crystal data

C_14_H_11_ClN_2_O_2_
*M* *_r_* = 274.70
Orthorhombic, 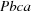

*a* = 11.8864 (4) Å
*b* = 13.6276 (5) Å
*c* = 31.0273 (10) Å
*V* = 5025.9 (3) Å^3^
*Z* = 16
Mo *K*α radiation
μ = 0.30 mm^−1^
*T* = 100 K
0.40 × 0.20 × 0.20 mm

#### Data collection

Agilent SuperNova Dual diffractometer with an Atlas detector
Absorption correction: multi-scan (*CrysAlis PRO*; Agilent, 2012 ▶) *T*_min_ = 0.817, *T*_max_ = 1.000
17994 measured reflections
5796 independent reflections
4522 reflections with *I* > 2σ(*I*)
*R*_int_ = 0.041

#### Refinement

*R*[*F* ^2^ > 2σ(*F* ^2^)] = 0.044
*wR*(*F* ^2^) = 0.117
*S* = 1.03
5796 reflections
347 parameters
H-atom parameters constrained
Δρ_max_ = 0.56 e Å^−3^
Δρ_min_ = −0.37 e Å^−3^

### 

Data collection: *CrysAlis PRO* (Agilent, 2012 ▶); cell refinement: *CrysAlis PRO*; data reduction: *CrysAlis PRO*; program(s) used to solve structure: *SHELXS97* (Sheldrick, 2008 ▶); program(s) used to refine structure: *SHELXL97* (Sheldrick, 2008 ▶); molecular graphics: *ORTEP-3* (Farrugia, 1997 ▶) and *DIAMOND* (Brandenburg, 2006 ▶); software used to prepare material for publication: *publCIF* (Westrip, 2010 ▶).

## Supplementary Material

Crystal structure: contains datablock(s) global, I. DOI: 10.1107/S1600536812028528/su2466sup1.cif

Structure factors: contains datablock(s) I. DOI: 10.1107/S1600536812028528/su2466Isup2.hkl

Supplementary material file. DOI: 10.1107/S1600536812028528/su2466Isup3.cml

Additional supplementary materials:  crystallographic information; 3D view; checkCIF report

## Figures and Tables

**Table 1:** Hydrogen-bond geometry (Å, °)

*D*—H⋯*A*	*D*—H	H⋯*A*	*D*⋯*A*	*D*—H⋯*A*
C12—H12⋯O2^i^	0.95	2.32	3.203 (2)	154
C14—H14⋯Cl2^i^	0.95	2.74	3.448 (2)	132

Symmetry code: (i) 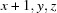.

**Fig. 1.**
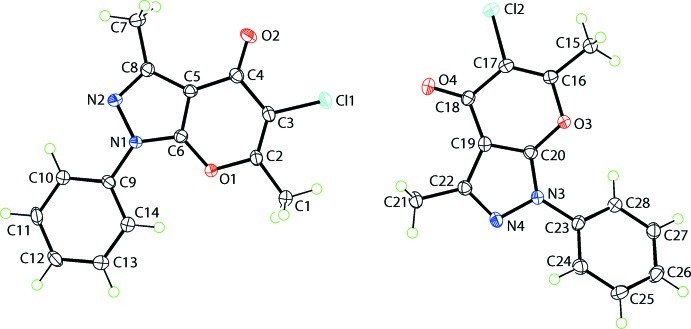
The molecular structure of the two independent molecules of (I), showing the atom-labelling scheme and displacement ellipsoids at the 50% probability level.

**Fig. 2.**
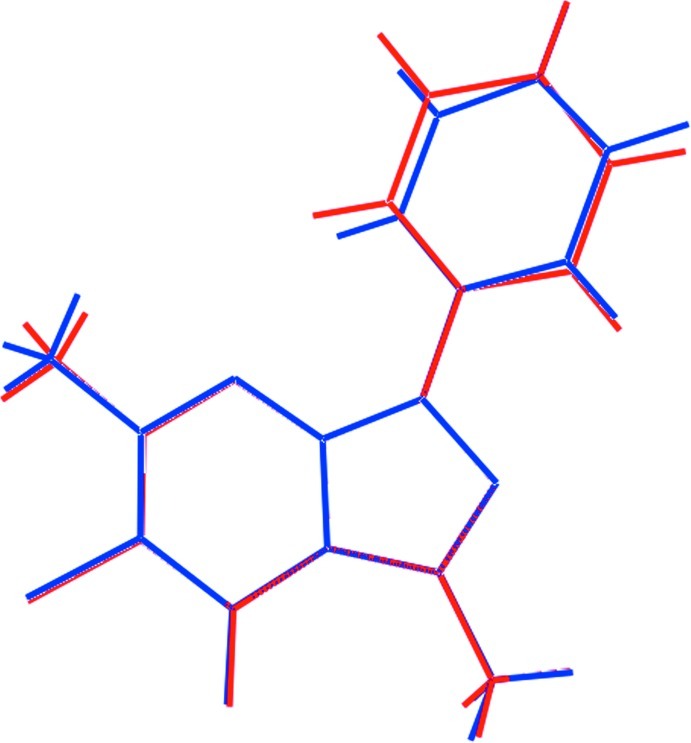
Super-imposition of the fused-ring systems of the two independent molecules in (I). The Cl1 and and Cl2-containing molecules are shown as red and blue images, respectively.

**Fig. 3.**
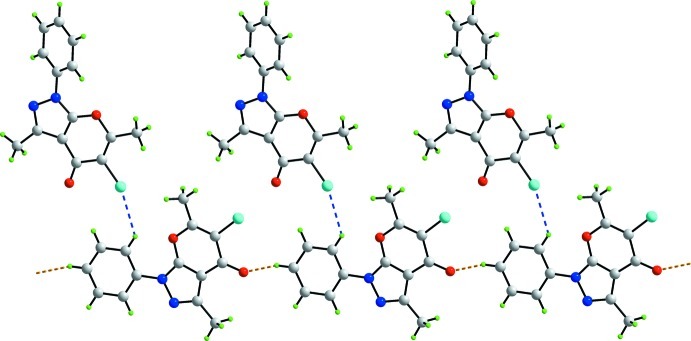
A view of the supramolecular chain along the a axis in (I) mediated by C—H···O and C—H···Cl interactions shown as orange and blue dashed lines, respectively.

**Fig. 4.**
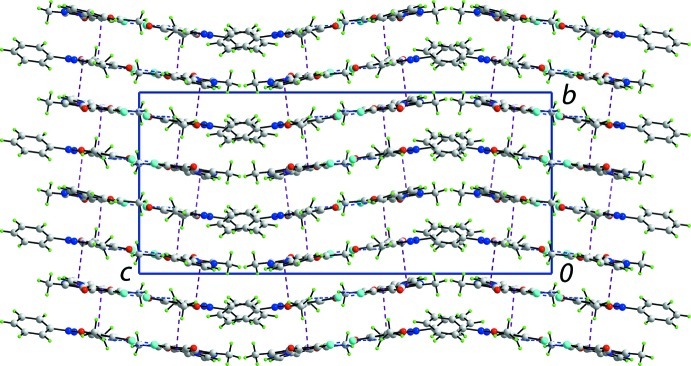
A view in projection down the a axis of the unit-cell contents of (I) highlighting the stacks of supramolecular layers along the c axis. The C—H···O, C—H···Cl and π—π interactions are shown as orange, blue and purple dashed lines, respectively.

## supplementary crystallographic information

### Comment

In connection with reports that pyrano[2,3-*c*]pyrazole derivatives possess analgesic and anti-inflammatory activities (Kuo *et al.*, 1984), the title compound (I) was synthesized, following a literature procedure (Gelin *et al.*, 1983), and its crystal and molecular structure are reported on herein.

In (I), Fig. 1, two independent molecules comprise the asymmetric unit. As seen from the overlay diagram, Fig. 2, these are virtually super-imposable. The primary difference between the molecules relates to the relative orientation of the pendent phenyl ring with respect to the fused-ring system [r.m.s. deviations = 0.024 and 0.021 Å, respectively] as seen in the dihedral angles of 8.32 (8) and 28.32 (8)°, respectively. In the structure of the derivative without a chloro substituent, the molecule is planar with the r.m.s. of all non-hydrogen atoms being 0.038 Å (Asiri *et al.*, 2012).

In the crystal, the Cl1-containing molecules are connected into a linear supramolecular chain along the *a* axis *via* C—H···O interactions and linked to this *via* C—H···Cl interactions are the Cl2-containing molecules, Fig. 3 and Table 1. Chains are connected into layers in the *ab* plane by π—π interactions with the closest of these occurring between the five-membered and six-membered in an alternating sequence of the independent molecules [ring centroid(N1-pyrazole)···(O3-pyrano)^i^ = 3.5442 (11) Å, angle of inclination = 2.29 (11)° for i: -x+1, -y, -z+1; ring centroid(N3-pyrazole)···(O1-pyrano)^ii^ = 3.4022 (10) Å, angle of inclination = 5.38 (8)° for ii: x+1/2, -y+1/2, -z+1]. The layers stack along the *c* axis with no specific intermolecular interactions between them, Fig. 4.

### Experimental

To a solution of 4-(acetoacetyl)-3-methyl-1-phenyl-2-pyrazolin-5-one (0.01 *M*), made following a literature procedure (Gelin *et al.*, 1983), in dry methylene chloride (20 ml) was added drop-wise sulfuryl chloride (1.35 g, 0.01 *M*). The mixture was allowed to stand at room temperature for 2 h and then poured into a 10% aqueous K_2_CO_3_ solution (50 ml) with stirring for 5 min. The aqueous layer was acidified with 10% HCl and extracted with chloroform. The combined organic extracts were washed with water and dried (Na_2_SO_4_). Removal of the solvent gave 4-(aceto-chloroacetyl)-3-methyl-1-phenyl-2-pyrazolin-5-one. Concentrated sulfuric acid (1 ml) was then added drop-wise. After 4 h at room temperature, the mixture was poured into ice-water (200 ml). The precipitate was extracted with chloroform. The chloroform layer was washed with 5% aqueous K_2_CO_3_ solution, dried and evaporated to give the title compound which was recrystallized from ethanol. M.p: 413–415 K *cf.* Lit. M.p. 413 K (Gelin *et al.*, 1983). Yield: 68%.

### Refinement

C-bound H-atoms were placed in calculated positions and included in the refinement in the riding model approximation: C—H = 0.95 and 0.98 Å for CH and CH_3_ H-atoms, respectively, with *U*_iso_(H) = k × U_eq_(C) where k = 1.5 for CH_3_ H-atoms and = 1.2 for other H-atoms.

### Crystal data

**Table:**

C_14_H_11_ClN_2_O_2_	*F*(000) = 2272
*M**_r_* = 274.70	*D*_x_ = 1.452 Mg m^−^^3^
Orthorhombic, *P**b**c**a*	Mo *K*α radiation, λ = 0.71073 Å
Hall symbol: -P 2ac 2ab	Cell parameters from 5991 reflections
*a* = 11.8864 (4) Å	θ = 2.3–27.5°
*b* = 13.6276 (5) Å	µ = 0.30 mm^−^^1^
*c* = 31.0273 (10) Å	*T* = 100 K
*V* = 5025.9 (3) Å^3^	Prism, colourless
*Z* = 16	0.40 × 0.20 × 0.20 mm

### Data collection

**Table:**

Agilent SuperNova Dual diffractometer with an Atlas detector	5796 independent reflections
Radiation source: SuperNova (Mo) X-ray Source	4522 reflections with *I* > 2σ(*I*)
Mirror monochromator	*R*_int_ = 0.041
Detector resolution: 10.4041 pixels mm^-1^	θ_max_ = 27.6°, θ_min_ = 2.4°
ω scan	*h* = −14→15
Absorption correction: multi-scan (*CrysAlis PRO*; Agilent, 2012)	*k* = −17→10
*T*_min_ = 0.817, *T*_max_ = 1.000	*l* = −23→40
17994 measured reflections	

### Refinement

**Table:**

Refinement on *F*^2^	Primary atom site location: structure-invariant direct methods
Least-squares matrix: full	Secondary atom site location: difference Fourier map
*R*[*F*^2^ > 2σ(*F*^2^)] = 0.044	Hydrogen site location: inferred from neighbouring sites
*wR*(*F*^2^) = 0.117	H-atom parameters constrained
*S* = 1.03	*w* = 1/[σ^2^(*F*_o_^2^) + (0.0556*P*)^2^ + 1.8272*P*] where *P* = (*F*_o_^2^ + 2*F*_c_^2^)/3
5796 reflections	(Δ/σ)_max_ = 0.001
347 parameters	Δρ_max_ = 0.56 e Å^−^^3^
0 restraints	Δρ_min_ = −0.37 e Å^−^^3^

### Special details

**Table:**

Geometry. All e.s.d.'s (except the e.s.d. in the dihedral angle between two l.s. planes) are estimated using the full covariance matrix. The cell e.s.d.'s are taken into account individually in the estimation of e.s.d.'s in distances, angles and torsion angles; correlations between e.s.d.'s in cell parameters are only used when they are defined by crystal symmetry. An approximate (isotropic) treatment of cell e.s.d.'s is used for estimating e.s.d.'s involving l.s. planes.
Refinement. Refinement of *F*^2^ against ALL reflections. The weighted *R*-factor *wR* and goodness of fit *S* are based on *F*^2^, conventional *R*-factors *R* are based on *F*, with *F* set to zero for negative *F*^2^. The threshold expression of *F*^2^ > σ(*F*^2^) is used only for calculating *R*-factors(gt) *etc*. and is not relevant to the choice of reflections for refinement. *R*-factors based on *F*^2^ are statistically about twice as large as those based on *F*, and *R*- factors based on ALL data will be even larger.

### Fractional atomic coordinates and isotropic or equivalent isotropic displacement parameters (Å^2^)

**Table:**

	*x*	*y*	*z*	*U*_iso_*/*U*_eq_	
Cl1	0.47784 (4)	0.10807 (4)	0.536902 (15)	0.02329 (13)	
Cl2	0.02904 (4)	0.13031 (4)	0.482067 (16)	0.02722 (14)	
O1	0.80047 (10)	0.08977 (9)	0.56939 (4)	0.0182 (3)	
O2	0.49310 (11)	0.08983 (10)	0.63216 (5)	0.0250 (3)	
O3	0.10832 (10)	0.18078 (9)	0.36014 (4)	0.0182 (3)	
O4	0.27633 (11)	0.13467 (10)	0.47565 (4)	0.0241 (3)	
N1	0.87843 (13)	0.06692 (12)	0.63947 (5)	0.0173 (3)	
N2	0.83780 (13)	0.05555 (11)	0.68112 (5)	0.0190 (3)	
N3	0.29023 (13)	0.18475 (11)	0.32886 (5)	0.0187 (3)	
N4	0.40146 (12)	0.17885 (12)	0.34252 (5)	0.0204 (3)	
C1	0.72337 (17)	0.10797 (16)	0.49990 (6)	0.0240 (4)	
H1A	0.6531	0.1003	0.4837	0.036*	
H1B	0.7769	0.0573	0.4909	0.036*	
H1C	0.7553	0.1730	0.4942	0.036*	
C2	0.70039 (15)	0.09791 (13)	0.54658 (6)	0.0186 (4)	
C3	0.59974 (15)	0.09712 (14)	0.56689 (6)	0.0192 (4)	
C4	0.58537 (15)	0.08820 (13)	0.61417 (6)	0.0186 (4)	
C5	0.69231 (15)	0.07793 (13)	0.63565 (6)	0.0168 (4)	
C6	0.79054 (15)	0.07976 (13)	0.61251 (6)	0.0164 (4)	
C7	0.65511 (17)	0.05340 (15)	0.71804 (6)	0.0237 (4)	
H7A	0.7022	0.0369	0.7429	0.035*	
H7B	0.5990	0.0017	0.7136	0.035*	
H7C	0.6169	0.1160	0.7233	0.035*	
C8	0.72680 (15)	0.06198 (13)	0.67899 (6)	0.0184 (4)	
C9	0.99726 (15)	0.06552 (13)	0.63192 (6)	0.0172 (4)	
C10	1.06841 (16)	0.04123 (15)	0.66587 (6)	0.0221 (4)	
H10	1.0387	0.0267	0.6936	0.027*	
C11	1.18377 (16)	0.03853 (16)	0.65855 (7)	0.0256 (4)	
H11	1.2330	0.0215	0.6815	0.031*	
C12	1.22826 (16)	0.06032 (15)	0.61829 (7)	0.0235 (4)	
H12	1.3072	0.0582	0.6136	0.028*	
C13	1.15581 (16)	0.08529 (15)	0.58498 (7)	0.0240 (4)	
H13	1.1856	0.1008	0.5574	0.029*	
C14	1.04043 (16)	0.08789 (15)	0.59154 (6)	0.0221 (4)	
H14	0.9913	0.1048	0.5686	0.027*	
C15	−0.07309 (16)	0.17796 (15)	0.39208 (6)	0.0216 (4)	
H15A	−0.1128	0.1366	0.4130	0.032*	
H15B	−0.0929	0.1574	0.3628	0.032*	
H15C	−0.0949	0.2466	0.3963	0.032*	
C16	0.05018 (16)	0.16775 (13)	0.39842 (6)	0.0181 (4)	
C17	0.10543 (16)	0.15005 (14)	0.43572 (6)	0.0189 (4)	
C18	0.22949 (16)	0.14778 (13)	0.44089 (6)	0.0176 (4)	
C19	0.28413 (15)	0.16257 (13)	0.39974 (6)	0.0177 (4)	
C20	0.22119 (15)	0.17546 (13)	0.36295 (6)	0.0169 (4)	
C21	0.50367 (16)	0.15383 (15)	0.41053 (7)	0.0240 (4)	
H21A	0.5689	0.1630	0.3916	0.036*	
H21B	0.5061	0.0880	0.4232	0.036*	
H21C	0.5053	0.2030	0.4336	0.036*	
C22	0.39769 (15)	0.16529 (13)	0.38481 (6)	0.0187 (4)	
C23	0.26647 (16)	0.19886 (13)	0.28403 (6)	0.0186 (4)	
C24	0.34540 (16)	0.24761 (14)	0.25905 (6)	0.0213 (4)	
H24	0.4129	0.2716	0.2716	0.026*	
C25	0.32434 (17)	0.26094 (15)	0.21537 (6)	0.0257 (4)	
H25	0.3779	0.2940	0.1979	0.031*	
C26	0.22545 (17)	0.22611 (15)	0.19720 (6)	0.0264 (5)	
H26	0.2114	0.2355	0.1673	0.032*	
C27	0.14728 (17)	0.17781 (15)	0.22247 (6)	0.0234 (4)	
H27	0.0796	0.1542	0.2099	0.028*	
C28	0.16698 (16)	0.16358 (15)	0.26614 (6)	0.0218 (4)	
H28	0.1134	0.1303	0.2835	0.026*	

### Atomic displacement parameters (Å^2^)

**Table:**

	*U*^11^	*U*^22^	*U*^33^	*U*^12^	*U*^13^	*U*^23^
Cl1	0.0166 (2)	0.0292 (3)	0.0240 (3)	0.00225 (19)	−0.00503 (18)	−0.0020 (2)
Cl2	0.0242 (3)	0.0396 (3)	0.0178 (2)	−0.0002 (2)	0.00323 (18)	0.0053 (2)
O1	0.0145 (6)	0.0254 (7)	0.0146 (7)	0.0014 (5)	−0.0003 (5)	−0.0012 (5)
O2	0.0145 (7)	0.0334 (8)	0.0270 (8)	0.0006 (6)	0.0026 (6)	−0.0034 (6)
O3	0.0147 (6)	0.0240 (7)	0.0161 (7)	0.0002 (5)	−0.0003 (5)	0.0024 (5)
O4	0.0233 (7)	0.0314 (8)	0.0178 (7)	0.0013 (6)	−0.0047 (5)	0.0031 (6)
N1	0.0139 (7)	0.0234 (8)	0.0146 (8)	−0.0006 (6)	0.0011 (6)	−0.0014 (6)
N2	0.0179 (8)	0.0240 (8)	0.0150 (8)	−0.0006 (7)	0.0027 (6)	−0.0019 (6)
N3	0.0155 (8)	0.0240 (8)	0.0165 (8)	0.0009 (6)	−0.0017 (6)	0.0012 (6)
N4	0.0155 (8)	0.0241 (8)	0.0215 (9)	−0.0006 (7)	−0.0035 (6)	0.0019 (7)
C1	0.0196 (10)	0.0339 (11)	0.0184 (10)	0.0001 (9)	−0.0028 (7)	−0.0002 (8)
C2	0.0164 (9)	0.0207 (9)	0.0187 (9)	0.0021 (8)	−0.0039 (7)	−0.0016 (8)
C3	0.0158 (9)	0.0207 (9)	0.0212 (10)	0.0018 (8)	−0.0041 (7)	−0.0031 (8)
C4	0.0171 (9)	0.0179 (9)	0.0209 (10)	−0.0010 (7)	−0.0010 (7)	−0.0025 (7)
C5	0.0154 (9)	0.0187 (9)	0.0164 (9)	−0.0006 (7)	0.0016 (7)	−0.0025 (7)
C6	0.0169 (9)	0.0165 (9)	0.0159 (9)	−0.0005 (7)	0.0007 (7)	−0.0023 (7)
C7	0.0213 (10)	0.0313 (11)	0.0184 (10)	−0.0020 (8)	0.0042 (8)	−0.0025 (8)
C8	0.0188 (9)	0.0186 (9)	0.0177 (10)	−0.0006 (7)	−0.0009 (7)	−0.0014 (7)
C9	0.0129 (9)	0.0189 (9)	0.0197 (10)	−0.0001 (7)	0.0006 (7)	−0.0040 (7)
C10	0.0175 (9)	0.0316 (11)	0.0172 (10)	−0.0002 (8)	−0.0008 (7)	−0.0026 (8)
C11	0.0179 (10)	0.0354 (11)	0.0237 (11)	0.0016 (9)	−0.0085 (8)	−0.0047 (9)
C12	0.0129 (9)	0.0293 (11)	0.0283 (11)	−0.0003 (8)	−0.0010 (8)	−0.0071 (9)
C13	0.0200 (10)	0.0310 (11)	0.0209 (10)	−0.0020 (8)	0.0022 (8)	−0.0007 (8)
C14	0.0196 (10)	0.0287 (10)	0.0182 (10)	0.0007 (8)	−0.0012 (7)	0.0009 (8)
C15	0.0183 (9)	0.0263 (10)	0.0201 (10)	−0.0006 (8)	−0.0008 (7)	0.0027 (8)
C16	0.0189 (9)	0.0180 (9)	0.0174 (9)	−0.0007 (7)	0.0013 (7)	−0.0008 (7)
C17	0.0204 (10)	0.0197 (9)	0.0167 (9)	−0.0009 (8)	0.0018 (7)	0.0011 (7)
C18	0.0203 (9)	0.0160 (8)	0.0166 (9)	0.0008 (7)	−0.0012 (7)	0.0001 (7)
C19	0.0177 (9)	0.0174 (9)	0.0180 (9)	0.0002 (7)	−0.0025 (7)	0.0008 (7)
C20	0.0163 (9)	0.0163 (8)	0.0182 (9)	0.0012 (7)	0.0005 (7)	0.0002 (7)
C21	0.0182 (10)	0.0300 (11)	0.0239 (11)	−0.0017 (8)	−0.0047 (8)	0.0028 (9)
C22	0.0174 (9)	0.0184 (9)	0.0202 (10)	−0.0014 (7)	−0.0008 (7)	0.0016 (7)
C23	0.0217 (9)	0.0194 (9)	0.0147 (9)	0.0046 (8)	−0.0012 (7)	0.0005 (7)
C24	0.0201 (10)	0.0232 (9)	0.0207 (10)	0.0016 (8)	−0.0002 (7)	−0.0022 (8)
C25	0.0272 (11)	0.0319 (11)	0.0181 (10)	0.0012 (9)	0.0058 (8)	0.0018 (8)
C26	0.0318 (11)	0.0321 (11)	0.0153 (10)	0.0069 (9)	0.0001 (8)	0.0002 (8)
C27	0.0216 (10)	0.0287 (10)	0.0197 (10)	0.0040 (8)	−0.0033 (8)	−0.0020 (8)
C28	0.0200 (10)	0.0268 (10)	0.0185 (10)	0.0013 (8)	0.0001 (7)	0.0022 (8)

### Geometric parameters (Å, º)

**Table:**

Cl1—C3	1.7285 (19)	C10—H10	0.9500
Cl2—C17	1.7218 (19)	C11—C12	1.389 (3)
O1—C6	1.350 (2)	C11—H11	0.9500
O1—C2	1.389 (2)	C12—C13	1.388 (3)
O2—C4	1.231 (2)	C12—H12	0.9500
O3—C20	1.346 (2)	C13—C14	1.387 (3)
O3—C16	1.386 (2)	C13—H13	0.9500
O4—C18	1.227 (2)	C14—H14	0.9500
N1—C6	1.350 (2)	C15—C16	1.485 (3)
N1—N2	1.388 (2)	C15—H15A	0.9800
N1—C9	1.432 (2)	C15—H15B	0.9800
N2—C8	1.324 (2)	C15—H15C	0.9800
N3—C20	1.345 (2)	C16—C17	1.352 (3)
N3—N4	1.391 (2)	C17—C18	1.484 (3)
N3—C23	1.432 (2)	C18—C19	1.447 (3)
N4—C22	1.326 (2)	C19—C20	1.376 (2)
C1—C2	1.480 (3)	C19—C22	1.428 (3)
C1—H1A	0.9800	C21—C22	1.499 (3)
C1—H1B	0.9800	C21—H21A	0.9800
C1—H1C	0.9800	C21—H21B	0.9800
C2—C3	1.352 (3)	C21—H21C	0.9800
C3—C4	1.482 (3)	C23—C24	1.386 (3)
C4—C5	1.442 (3)	C23—C28	1.392 (3)
C5—C6	1.371 (2)	C24—C25	1.390 (3)
C5—C8	1.423 (3)	C24—H24	0.9500
C7—C8	1.486 (3)	C25—C26	1.387 (3)
C7—H7A	0.9800	C25—H25	0.9500
C7—H7B	0.9800	C26—C27	1.382 (3)
C7—H7C	0.9800	C26—H26	0.9500
C9—C14	1.388 (3)	C27—C28	1.389 (3)
C9—C10	1.391 (3)	C27—H27	0.9500
C10—C11	1.390 (3)	C28—H28	0.9500
			
C6—O1—C2	115.99 (14)	C14—C13—H13	119.6
C20—O3—C16	115.74 (14)	C12—C13—H13	119.6
C6—N1—N2	108.79 (14)	C13—C14—C9	119.50 (18)
C6—N1—C9	131.59 (16)	C13—C14—H14	120.2
N2—N1—C9	119.60 (14)	C9—C14—H14	120.2
C8—N2—N1	107.03 (15)	C16—C15—H15A	109.5
C20—N3—N4	109.56 (15)	C16—C15—H15B	109.5
C20—N3—C23	131.01 (16)	H15A—C15—H15B	109.5
N4—N3—C23	119.42 (15)	C16—C15—H15C	109.5
C22—N4—N3	106.12 (15)	H15A—C15—H15C	109.5
C2—C1—H1A	109.5	H15B—C15—H15C	109.5
C2—C1—H1B	109.5	C17—C16—O3	120.95 (16)
H1A—C1—H1B	109.5	C17—C16—C15	127.54 (17)
C2—C1—H1C	109.5	O3—C16—C15	111.49 (15)
H1A—C1—H1C	109.5	C16—C17—C18	125.37 (17)
H1B—C1—H1C	109.5	C16—C17—Cl2	119.11 (15)
C3—C2—O1	121.32 (17)	C18—C17—Cl2	115.51 (14)
C3—C2—C1	128.32 (17)	O4—C18—C19	126.34 (18)
O1—C2—C1	110.36 (15)	O4—C18—C17	123.30 (17)
C2—C3—C4	124.33 (17)	C19—C18—C17	110.36 (16)
C2—C3—Cl1	119.35 (15)	C20—C19—C22	103.99 (16)
C4—C3—Cl1	116.32 (14)	C20—C19—C18	120.39 (17)
O2—C4—C5	125.28 (18)	C22—C19—C18	135.59 (17)
O2—C4—C3	123.38 (17)	O3—C20—N3	123.50 (16)
C5—C4—C3	111.33 (16)	O3—C20—C19	127.05 (17)
C6—C5—C8	104.63 (16)	N3—C20—C19	109.44 (16)
C6—C5—C4	120.46 (17)	C22—C21—H21A	109.5
C8—C5—C4	134.88 (17)	C22—C21—H21B	109.5
O1—C6—N1	124.03 (16)	H21A—C21—H21B	109.5
O1—C6—C5	126.54 (16)	C22—C21—H21C	109.5
N1—C6—C5	109.41 (16)	H21A—C21—H21C	109.5
C8—C7—H7A	109.5	H21B—C21—H21C	109.5
C8—C7—H7B	109.5	N4—C22—C19	110.89 (16)
H7A—C7—H7B	109.5	N4—C22—C21	120.85 (17)
C8—C7—H7C	109.5	C19—C22—C21	128.26 (17)
H7A—C7—H7C	109.5	C24—C23—C28	121.18 (17)
H7B—C7—H7C	109.5	C24—C23—N3	118.27 (17)
N2—C8—C5	110.14 (16)	C28—C23—N3	120.55 (17)
N2—C8—C7	121.71 (17)	C23—C24—C25	119.05 (18)
C5—C8—C7	128.14 (17)	C23—C24—H24	120.5
C14—C9—C10	120.74 (17)	C25—C24—H24	120.5
C14—C9—N1	120.63 (16)	C26—C25—C24	120.27 (19)
C10—C9—N1	118.63 (17)	C26—C25—H25	119.9
C11—C10—C9	118.84 (18)	C24—C25—H25	119.9
C11—C10—H10	120.6	C27—C26—C25	120.13 (19)
C9—C10—H10	120.6	C27—C26—H26	119.9
C12—C11—C10	121.11 (18)	C25—C26—H26	119.9
C12—C11—H11	119.4	C26—C27—C28	120.43 (19)
C10—C11—H11	119.4	C26—C27—H27	119.8
C11—C12—C13	119.08 (18)	C28—C27—H27	119.8
C11—C12—H12	120.5	C27—C28—C23	118.95 (18)
C13—C12—H12	120.5	C27—C28—H28	120.5
C14—C13—C12	120.71 (19)	C23—C28—H28	120.5
			
C6—N1—N2—C8	−0.26 (19)	C10—C9—C14—C13	0.4 (3)
C9—N1—N2—C8	178.57 (16)	N1—C9—C14—C13	−179.52 (17)
C20—N3—N4—C22	0.45 (19)	C20—O3—C16—C17	−0.6 (2)
C23—N3—N4—C22	−179.61 (16)	C20—O3—C16—C15	178.27 (15)
C6—O1—C2—C3	0.9 (2)	O3—C16—C17—C18	3.4 (3)
C6—O1—C2—C1	−179.75 (15)	C15—C16—C17—C18	−175.20 (18)
O1—C2—C3—C4	0.3 (3)	O3—C16—C17—Cl2	−177.84 (13)
C1—C2—C3—C4	−178.98 (18)	C15—C16—C17—Cl2	3.5 (3)
O1—C2—C3—Cl1	179.71 (13)	C16—C17—C18—O4	177.47 (18)
C1—C2—C3—Cl1	0.5 (3)	Cl2—C17—C18—O4	−1.3 (2)
C2—C3—C4—O2	178.32 (18)	C16—C17—C18—C19	−2.8 (3)
Cl1—C3—C4—O2	−1.1 (2)	Cl2—C17—C18—C19	178.44 (13)
C2—C3—C4—C5	−1.5 (3)	O4—C18—C19—C20	179.30 (18)
Cl1—C3—C4—C5	179.05 (13)	C17—C18—C19—C20	−0.4 (2)
O2—C4—C5—C6	−178.18 (18)	O4—C18—C19—C22	1.6 (3)
C3—C4—C5—C6	1.6 (2)	C17—C18—C19—C22	−178.2 (2)
O2—C4—C5—C8	4.2 (3)	C16—O3—C20—N3	178.86 (16)
C3—C4—C5—C8	−176.02 (19)	C16—O3—C20—C19	−2.8 (3)
C2—O1—C6—N1	177.20 (16)	N4—N3—C20—O3	178.15 (15)
C2—O1—C6—C5	−0.7 (3)	C23—N3—C20—O3	−1.8 (3)
N2—N1—C6—O1	−177.71 (15)	N4—N3—C20—C19	−0.4 (2)
C9—N1—C6—O1	3.6 (3)	C23—N3—C20—C19	179.65 (17)
N2—N1—C6—C5	0.5 (2)	C22—C19—C20—O3	−178.29 (17)
C9—N1—C6—C5	−178.13 (18)	C18—C19—C20—O3	3.3 (3)
C8—C5—C6—O1	177.63 (16)	C22—C19—C20—N3	0.2 (2)
C4—C5—C6—O1	−0.6 (3)	C18—C19—C20—N3	−178.16 (16)
C8—C5—C6—N1	−0.5 (2)	N3—N4—C22—C19	−0.3 (2)
C4—C5—C6—N1	−178.81 (16)	N3—N4—C22—C21	178.95 (16)
N1—N2—C8—C5	−0.1 (2)	C20—C19—C22—N4	0.1 (2)
N1—N2—C8—C7	−179.79 (16)	C18—C19—C22—N4	178.07 (19)
C6—C5—C8—N2	0.4 (2)	C20—C19—C22—C21	−179.13 (18)
C4—C5—C8—N2	178.28 (19)	C18—C19—C22—C21	−1.1 (3)
C6—C5—C8—C7	−179.94 (18)	C20—N3—C23—C24	152.03 (19)
C4—C5—C8—C7	−2.0 (3)	N4—N3—C23—C24	−27.9 (2)
C6—N1—C9—C14	6.0 (3)	C20—N3—C23—C28	−28.5 (3)
N2—N1—C9—C14	−172.56 (16)	N4—N3—C23—C28	151.54 (17)
C6—N1—C9—C10	−173.99 (18)	C28—C23—C24—C25	−0.2 (3)
N2—N1—C9—C10	7.5 (2)	N3—C23—C24—C25	179.24 (17)
C14—C9—C10—C11	−0.8 (3)	C23—C24—C25—C26	0.2 (3)
N1—C9—C10—C11	179.18 (17)	C24—C25—C26—C27	−0.1 (3)
C9—C10—C11—C12	0.5 (3)	C25—C26—C27—C28	−0.1 (3)
C10—C11—C12—C13	0.1 (3)	C26—C27—C28—C23	0.1 (3)
C11—C12—C13—C14	−0.5 (3)	C24—C23—C28—C27	0.0 (3)
C12—C13—C14—C9	0.2 (3)	N3—C23—C28—C27	−179.40 (17)

### Hydrogen-bond geometry (Å, º)

**Table:**

*D*—H···*A*	*D*—H	H···*A*	*D*···*A*	*D*—H···*A*
C12—H12···O2^i^	0.95	2.32	3.203 (2)	154
C14—H14···Cl2^i^	0.95	2.74	3.448 (2)	132

Symmetry code: (i) *x*+1, *y*, *z*.
